# Evaluating T1/T2 Relaxometry with OCRA Tabletop MRI System in Fresh Clinical Samples: Preliminary Insights into ZEB1-Associated Tissue Characteristics

**DOI:** 10.1177/15330338251366371

**Published:** 2025-08-26

**Authors:** Ahmed Y. Sanin, Marcus Prier, Thomas Wartmann, Christian Siba, Katrin Hippe, Maciej Pech, Roland S. Croner, Oliver Speck, Ulf D. Kahlert, Georg Rose

**Affiliations:** 1 Molecular and Experimental Surgery, Medical Faculty University Hospital Magdeburg, University Clinic for General, Visceral, Vascular and Transplantation Surgery, 9376 Otto-von Guericke University, Magdeburg, Germany; 2 Research Campus STIMULATE, 9376 Otto-von Guericke University, Magdeburg, Germany; 3 Institute of Physics, Otto-von Guericke University, Magdeburg, Germany; 4 Institute for Pathology, 9376 Otto-von Guericke University, Magdeburg, Germany; 5 Department of Radiology and Nuclear Medicine (KRN), Otto-von Guericke University, Magdeburg, Germany; 6 Institute of Medical Technology, Otto-von Guericke University, Magdeburg, Germany

**Keywords:** MRI, relaxometry, EMT, tumor microenvironment

## Abstract

**Introduction:**

The OCRA Tabletop MRI System is a compact, low-field (0.24T) magnetic resonance platform originally developed as an educational device to teach MR physics using chemical test tube–sized samples. Given its capabilities, we explored its diagnostic potential by performing relaxometric analysis on freshly resected human tissue specimens.

**Methods:**

Matched pairs of histologically confirmed tumor and non-tumor samples were analyzed with the OCRA MRI system to determine T1 and T2 relaxation times via NMR spectroscopy. In parallel, mRNA expression levels of ZEB1, a key transcription factor involved in WNT signaling, stem cell maintenance and tumor–stroma interactions were quantified for each sample.

**Results:**

The measured T1 and T2 relaxation times showed distinct profiles between tumor and non-tumor tissues. These biophysical properties were correlated with ZEB1 mRNA expression, revealing preliminary associations between tissue relaxation behavior and molecular signatures relevant to tumor microenvironment dynamics.

**Conclusion:**

Although this pilot study does not yet confirm clinical diagnostic utility, it offers initial biophysical insights into tumor–associated tissue alterations and provides a foundation for future validation studies in larger patient cohorts.

## Introduction

Magnetic Resonance Imaging (MRI) has long been an essential tool for both medical diagnostics and research applications, offering detailed insights into tissue composition and function. The OCRA Tabletop MRI System,^1,2^ is a compact MRI system designed for spectroscopy and imaging of small test tube-sized samples. The spin-lattice relaxation time T1 of a sample can be evaluated with an inversion recovery sequence by sweeping the inversion time TI and the spin-spin relaxation time T2 can be evaluated with a spin-echo sequence by sweeping the echo time TE. Both times are influenced by key tissue properties, including water content, cellular density and elasticity.

**Figure 1.**
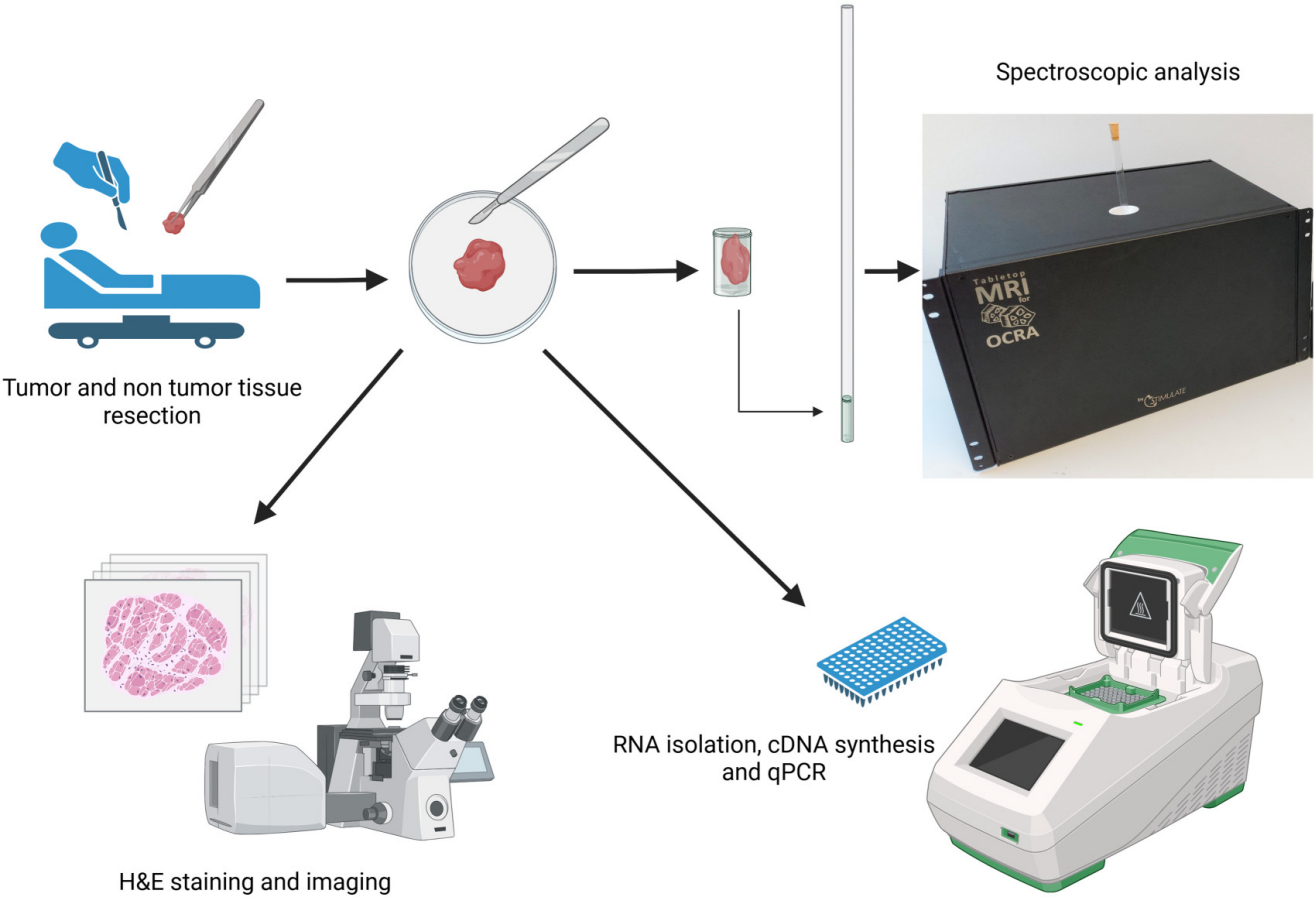
Schematic Representation of Experimental OCRA Tabletop MRI System's Workflow for Relaxation Based Characterization. The Pipeline Outlines Sample Acquisition, Tissue Preparation, MRI Scanning Using the System, Followed by Computational Analysis of Relaxometric and Transcriptional Regulator Profiling of Tumor-non Tumor Regions.

Higher water content (eg, CSF, edema) leads to longer T1 and T2, while low water content in fibrotic or dense tissues results in shorter relaxation times.^3^ Magnetic relaxation analysis demonstrates that increased macromolecular rigidity and reduced internal motion significantly restrict water proton mobility, resulting in shortened T1 and T2 relaxation time.^4^ Similarly, elastic tissues (eg, normal liver, muscle) exhibit longer T1 and T2, while stiff, fibrotic tissues (eg, desmoplastic tumors) display shortened values due to extracellular matrix (ECM) remodeling.^5,6^ These MRI characteristics provide critical insights into tissue composition, tumor microenvironments and pathological alterations. An overview of the experimental workflow and analytical steps used in this study is illustrated in Figure 1. Furthermore, the investigation of ZEB1 mediated epithelial-to-mesenchymal transition (EMT) induced tissue alterations and regulation of the WNT signalling pathway, which is crucial for stem cell maintenance, offering non-invasive insights into tumor progression and stromal interactions.^7,8^

## Materials and Methods

Informed consent for the procurement of surgically resected tissues was obtained from patients ≥7 days prior to elective procedures. Immediately following sample acquisition, each specimen was assigned a unique identifier and anonymized. Patient-specific information was securely stored on a restricted high-security server, ensuring that all samples remained de-identified and patient confidentiality was fully maintained. Collected samples were preserved in Advanced DMEM/F12 culture medium (Gibco, #12634010) supplemented with 1% GlutaMAX (Thermo Fisher, #35050038) and 1% penicillin-streptomycin (Thermo Fisher, #15140122) to maintain tissue viability and minimize microbial contamination. All the tissues were brought to the scanning facility after 14 h in average and the segments (∼10mm³) were embedded into 3D printed sample holder (PLA) inside a chemical test tube to place the sample into the iso center of the Tabletop MRI. The relaxation measurements were evaluated three times to give an average and error bar for the relaxation times (Supplementary Table 1). A consistent imaging protocol was applied across all replicates, with identical acquisition parameters used for each tumor tissue measurement and a separate, uniform parameter set employed for all non-tumor tissue replicates (Supplementary Table 2). While some spectroscopy parameters like the sampling time can be set to a global value, the sweeping parameters were adjusted to fit the individual sample to match the relaxation times. For T2 mapping, a monoexponential decay model, *S(TE) ∼e^(–TE/T^*^2),^ was linearized via logarithmic transformation, and T2 was calculated as the reciprocal of the negative slope obtained from standard linear regression. For T1 mapping, an inversion recovery model, S_MAX_ - *S(TI) ∼ -e^(–TI/T1)^)*, was used, where initial data points were inverted around up to the zero crossing and the recovery curve was linearized, with T1 determined as the reciprocal of the fitted slope. The highest data point (long TI) was used as the maximum signal S_MAX_.^9^

Parallel cryopreserved tissue aliquots stabilized in RNAlater (invitrogen, #10427114) were mechanically homogenized, followed by total RNA isolation via the ReliaPrep™ miRNA Cell and Tissue Miniprep System (Promega, #Z6212), adhering to manufacturer's instructions. RNA was reverse-transcribed using LunaScript^®^ RT SuperMix (NEB, #M3010L). ZEB1 mRNA expression was quantified via Luna Universal qPCR Master Mix (NEB) with primers (Microsynth AG) targeting ZEB1 (forward: 5′- CGA ACC CGC GGC GCA ATA-3′; reverse: 5′- CCA GCA GTT CTT GCA ATT CC-3′) and normalized to β-actin (housekeeping control).

## Result

**Figure 2.**
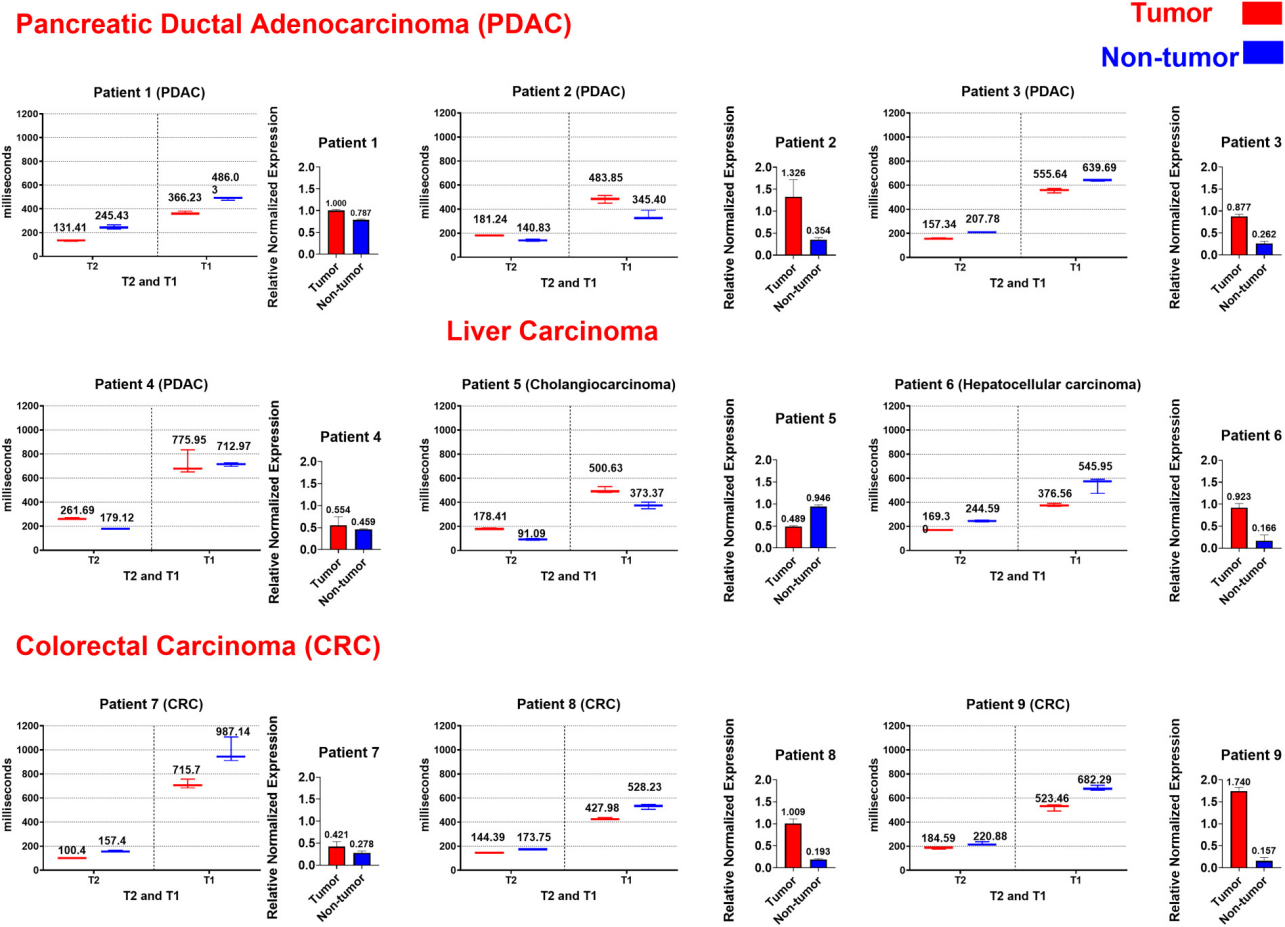
Relaxometric and ZEB1 Transcriptional Regulator Profiling of the Matching Paired Tissue Samples, Featuring Pancreatic Ductal Adenocarcinoma (PDAC), Colorectal Carcinoma (CRC) and Liver Carcinoma (Hepatocellular and Cholangiocarcinoma). For the Relaxometric plots T1 and T2 Relaxation Time at X Axis and T1 was Extracted by Fitting the Signal as a Function of Inversion Time (TI), and T2 was Obtained from Signal Decay Curves Measured at Multiple Echo Times (TE). In the Gene Expression Plots Tissue Types are at X Axis and Relative Normalized Gene Expression (ΔΔCt) is at Y Axis, with Patient 1 tumor used as the reference (set to 1.000) for comparative expression analysis.

**Figure 3.**
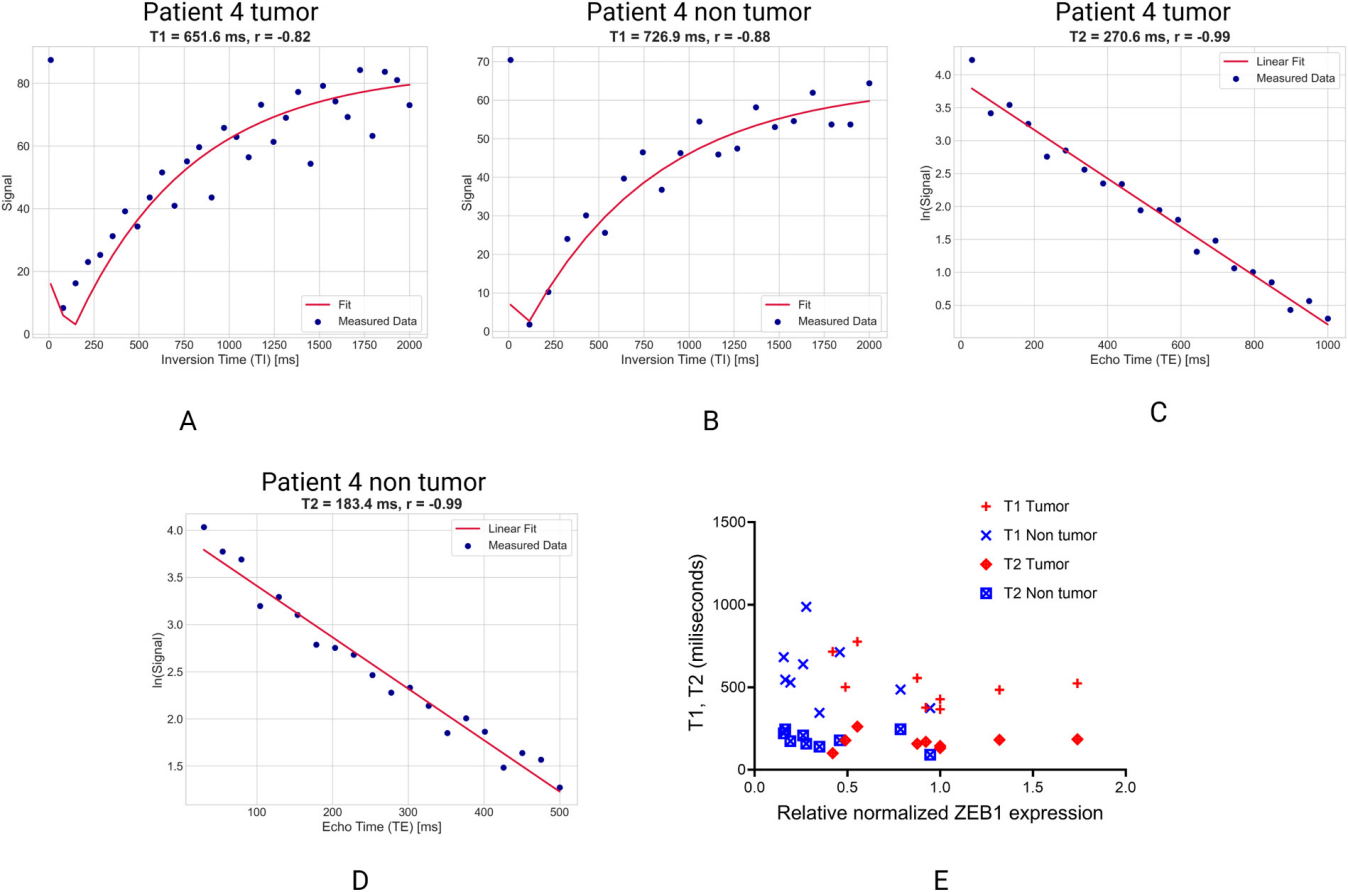
Representative raw Signal Plots from Patient 4 Showing Measured T1 and T2 Relaxation Times, Alongside the Relative Expression of ZEB1. Panels A and B Show T1 Relaxation Curves Plotted as Signal Intensity Versus Inversion Time (TI), While Panels C and D Depict T2 Relaxation Analysis as the Natural Logarithm of Signal Intensity Versus echo Time (TE). Panel E Illustrates the Relationship Between ZEB1 Expression Levels and Corresponding T1 and T2 Relaxation Times for Individual Samples.

Across most patients, tumor tissues (PDAC, CRC and liver carcinoma) exhibited significantly reduced T1 and T2 relaxation times compared to matched non-tumor tissues (Figure 2). This pattern was consistent in 6 out of 9 patients (Patients 1, 3, 6, 7, 8, 9), with tumors showing lower T1 (12-45% reduction) and lower T2 (22-55% reduction). Tumors with low ZEB1 expression (eg, Patient 5: ZEB1 = 0.4894) exhibited the shortest T1/T2 values, aligning with possibility of hypercellular/fibrotic phenotypes. Such exceptions included Patient 2 (PDAC), patient 4 (PDAC) and Patient 5 (liver carcinoma), where non-tumor T1/T2 values were paradoxically lower than tumor values, likely reflecting decreased stromal characteristics (eg, edema, inflammation). Notably, an inverse relationship was observed between ZEB1 expression and relaxation times, suggesting that increased transcriptional activity of ZEB1 may be associated with biophysical tissue changes indicative of reduced water mobility and higher rigidity (Figure 3)

## Discussion

The inverse correlation between ZEB1 expression and T1/T2 relaxation times in tumors reflects ZEB1's dual role in driving EMT and stromal remodeling. Elevated ZEB1 in tumors promotes hypercellularity via proliferative signalling and fibrosis through TGF-β/MMP-mediated collagen deposition by cancer-associated fibroblasts, restricting intracellular water mobility which can possibly decrease T2 and enhance spin-spin interactions shortening T1.^10^ Conversely, adjacent tumor cell free tissues with low ZEB1 exhibit prolonged T1/T2 due to tumor-secreted VEGF leading to an induction of angiogenesis and formation edema, thus increasing free water content.^11^ These ZEB1-associated changes in T1/T2 relaxation times suggest a possible link between molecular alterations and tissue biophysics. While these findings are preliminary, they demonstrate that the OCRA Tabletop MRI System may serve as a complementary tool for exploratory relaxometric profiling in research settings. Exceptions, such as elevated ZEB1 in non-tumor regions (eg, Patient 5), may arise from pre-malignant tissue areas or regions with epithelial-mesenchymal plasticity unrelated to the primary tumor or chronic inflammation in non-tumor regions, causing a transient upregulation of ZEB1, mimicking the tumorigenic molecular composition of EMT.

One limitation of this study is the inability to use identical tissue sections for all experimental procedures-tabletop MRI, qPCR, and histological analysis (H&E staining), which would have ensured data homogeneity. However, operational constraints, such as the need to preserve tissue integrity for RNA extraction and histology, necessitated the use of separate sections. While tumors exhibit intratumoral heterogeneity, tissue selection was guided by pathologists, who classified samples as either tumor or tumor free regions based on H&E staining (Supplementary Figure 1). A balanced representation of demographic factors (age and gender) further reinforced the robustness of the experimental design (Supplementary Table 3). This approach ensured a balance between consistency and potential variations in tissue composition. Subsequent experimental data, however, demonstrate that the OCRA Tabletop MRI System successfully detected ZEB1 activation correlated with modified liquid properties within tumor tissue microenvironments.

## Supplemental Material

sj-tif-1-tct-10.1177_15330338251366371 - Supplemental material for Evaluating T1/T2 Relaxometry with OCRA Tabletop MRI System in Fresh Clinical Samples: Preliminary Insights into ZEB1-Associated Tissue Characteristics Supplemental material, sj-tif-1-tct-10.1177_15330338251366371 for Evaluating T1/T2 Relaxometry with OCRA Tabletop MRI System in Fresh Clinical Samples: Preliminary Insights into ZEB1-Associated Tissue Characteristics by Ahmed Y. Sanin, Marcus Prier, Thomas Wartmann and Christian Siba, Katrin Hippe, Maciej Pech, Roland S. Croner, Oliver Speck, Ulf D. Kahlert, Georg Rose in Technology in Cancer Research & Treatment

sj-docx-2-tct-10.1177_15330338251366371 - Supplemental material for Evaluating T1/T2 Relaxometry with OCRA Tabletop MRI System in Fresh Clinical Samples: Preliminary Insights into ZEB1-Associated Tissue Characteristics Supplemental material, sj-docx-2-tct-10.1177_15330338251366371 for Evaluating T1/T2 Relaxometry with OCRA Tabletop MRI System in Fresh Clinical Samples: Preliminary Insights into ZEB1-Associated Tissue Characteristics by Ahmed Y. Sanin, Marcus Prier, Thomas Wartmann and Christian Siba, Katrin Hippe, Maciej Pech, Roland S. Croner, Oliver Speck, Ulf D. Kahlert, Georg Rose in Technology in Cancer Research & Treatment

sj-docx-3-tct-10.1177_15330338251366371 - Supplemental material for Evaluating T1/T2 Relaxometry with OCRA Tabletop MRI System in Fresh Clinical Samples: Preliminary Insights into ZEB1-Associated Tissue Characteristics Supplemental material, sj-docx-3-tct-10.1177_15330338251366371 for Evaluating T1/T2 Relaxometry with OCRA Tabletop MRI System in Fresh Clinical Samples: Preliminary Insights into ZEB1-Associated Tissue Characteristics by Ahmed Y. Sanin, Marcus Prier, Thomas Wartmann and Christian Siba, Katrin Hippe, Maciej Pech, Roland S. Croner, Oliver Speck, Ulf D. Kahlert, Georg Rose in Technology in Cancer Research & Treatment

sj-docx-4-tct-10.1177_15330338251366371 - Supplemental material for Evaluating T1/T2 Relaxometry with OCRA Tabletop MRI System in Fresh Clinical Samples: Preliminary Insights into ZEB1-Associated Tissue Characteristics Supplemental material, sj-docx-4-tct-10.1177_15330338251366371 for Evaluating T1/T2 Relaxometry with OCRA Tabletop MRI System in Fresh Clinical Samples: Preliminary Insights into ZEB1-Associated Tissue Characteristics by Ahmed Y. Sanin, Marcus Prier, Thomas Wartmann and Christian Siba, Katrin Hippe, Maciej Pech, Roland S. Croner, Oliver Speck, Ulf D. Kahlert, Georg Rose in Technology in Cancer Research & Treatment

sj-docx-5-tct-10.1177_15330338251366371 - Supplemental material for Evaluating T1/T2 Relaxometry with OCRA Tabletop MRI System in Fresh Clinical Samples: Preliminary Insights into ZEB1-Associated Tissue Characteristics Supplemental material, sj-docx-5-tct-10.1177_15330338251366371 for Evaluating T1/T2 Relaxometry with OCRA Tabletop MRI System in Fresh Clinical Samples: Preliminary Insights into ZEB1-Associated Tissue Characteristics by Ahmed Y. Sanin, Marcus Prier, Thomas Wartmann and Christian Siba, Katrin Hippe, Maciej Pech, Roland S. Croner, Oliver Speck, Ulf D. Kahlert, Georg Rose in Technology in Cancer Research & Treatment
