# Supplementary figures and images for: Evaluating T1/T2 Relaxometry with OCRA Tabletop MRI System in Fresh Clinical Samples: Preliminary Insights into ZEB1-Associated Tissue Characteristics
Source: Technol Cancer Res Treat. 2025 Aug 26;24:15330338251366371. doi: 10.1177/15330338251366371 (PMC12381451; doi:10.1177/15330338251366371)

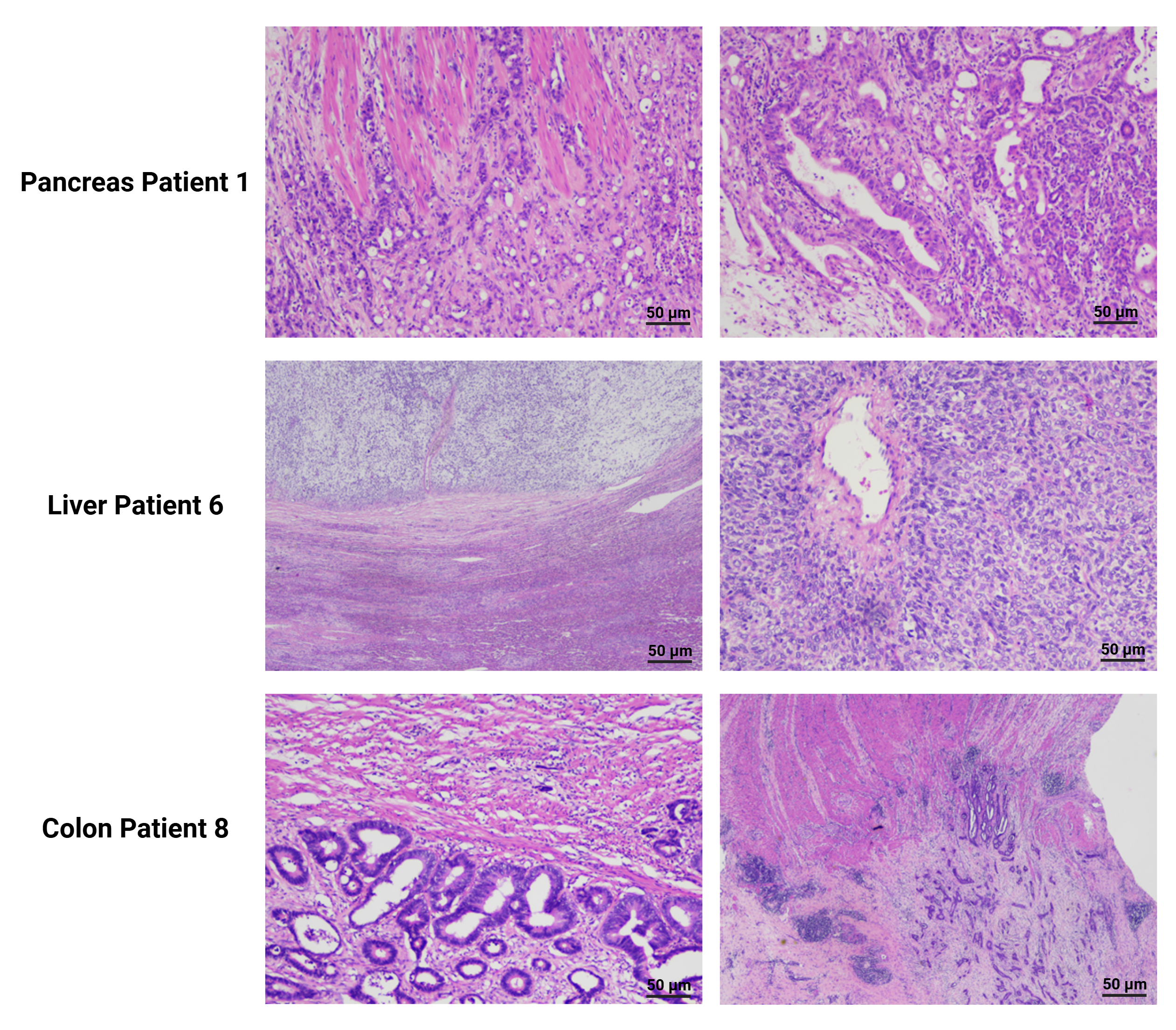

Supplement: sj-tif-1-tct-10.1177_15330338251366371 - Supplemental material for Evaluating T1/T2 Relaxometry with OCRA Tabletop MRI System in Fresh Clinical Samples: Preliminary Insights into ZEB1-Associated Tissue Characteristics [file sj-tif-1-tct-10.1177_15330338251366371.tif]
