# Supplementary material for: Evaluating T1/T2 Relaxometry with OCRA Tabletop MRI System in Fresh Clinical Samples: Preliminary Insights into ZEB1-Associated Tissue Characteristics
Source: Technol Cancer Res Treat. 2025 Aug 26;24:15330338251366371. doi: 10.1177/15330338251366371 (PMC12381451; doi:10.1177/15330338251366371)
Supplement: sj-docx-2-tct-10.1177_15330338251366371 - Supplemental material for Evaluating T1/T2 Relaxometry with OCRA Tabletop MRI System in Fresh Clinical Samples: Preliminary Insights into ZEB1-Associated Tissue Characteristics [file sj-docx-2-tct-10.1177_15330338251366371.docx]

| Sample details | | | T1 | | T2 | |
| --- | --- | --- | --- | --- | --- | --- |
|  |  |  | Relaxation time | R value | Relaxation time | R value |
| 1 | Tumor | Rep. 1 | 359.13 | -0.92 | 134.23 | -0.99 |
|  |  | Rep. 2 | 359.27 | -0.91 | 134.81 | -1.00 |
|  |  | Rep. 3 | 380.27 | -0.97 | 125.20 | -0.99 |
|  | Non-tumor | Rep. 1 | 471.92 | -0.95 | 242.30 | -1.00 |
|  |  | Rep. 2 | 492.56 | -0.97 | 229.05 | -0.99 |
|  |  | Rep. 3 | 493.61 | -0.96 | 264.95 | -1.00 |
| 2 | Tumor | Rep. 1 | 514.98 | -0.87 | 181.48 | -1.00 |
|  |  | Rep. 2 | 450.54 | -0.87 | 178.32 | -1.00 |
|  |  | Rep. 3 | 486.04 | -0.88 | 183.93 | -1.00 |
|  | Non-tumor | Rep. 1 | 389.91 | -0.89 | 132.74 | -1.00 |
|  |  | Rep. 2 | 320.28 | -0.90 | 150.57 | -1.00 |
|  |  | Rep. 3 | 326.02 | -0.85 | 139.20 | -0.99 |
| 3 | Tumor | Rep. 1 | 534.53 | -0.94 | 155.46 | -0.99 |
|  |  | Rep. 2 | 560.19 | -0.96 | 160.88 | -1.00 |
|  |  | Rep. 3 | 572.20 | -0.94 | 155.69 | -0.99 |
|  | Non-tumor | Rep. 1 | 632.71 | -0.95 | 205.15 | -1.00 |
|  |  | Rep. 2 | 643.62 | -0.95 | 209.73 | -0.99 |
|  |  | Rep. 3 | 642.74 | -0.94 | 208.47 | -0.99 |
| 4 | Tumor | Rep. 1 | 651.6 | -0.82 | 270.00 | -0.99 |
|  |  | Rep. 2 | 676.81 | -0.91 | 254.97 | -0.99 |
|  |  | Rep. 3 | 834.94 | -0.89 | 260.10 | -0.99 |
|  | Non-tumor | Rep. 1 | 726.93 | -0.88 | 183.37 | -0.99 |
|  |  | Rep. 2 | 715.00 | -0.82 | 175.37 | -0.99 |
|  |  | Rep. 3 | 697.00 | -0.87 | 178.63 | -0.99 |
| 5 | Tumor | Rep. 1 | 530.83 | -0.86 | 178.53 | -0.99 |
|  |  | Rep. 2 | 490.80 | -0.84 | 185.88 | -1.00 |
|  |  | Rep. 3 | 480.26 | -0.88 | 170.82 | -1.00 |
|  | Non-tumor | Rep. 1 | 374.27 | -0.82 | 84.74 | -1.00 |
|  |  | Rep. 2 | 345.61 | -0.84 | 91.06 | -0.99 |
|  |  | Rep. 3 | 400.25 | -0.85 | 97.47 | -0.99 |
| 6 | Tumor | Rep. 1 | 373.29 | -0.92 | 169.65 | -1.00 |
|  |  | Rep. 2 | 365.20 | -0.90 | 170.92 | -1.00 |
|  |  | Rep. 3 | 391.20 | -0.89 | 167.34 | -1.00 |
|  | Non-tumor | Rep. 1 | 474.09 | -0.93 | 235.94 | -0.99 |
|  |  | Rep. 2 | 590.51 | -0.90 | 244.79 | -0.99 |
|  |  | Rep. 3 | 573.24 | -0.88 | 253.06 | -0.99 |
| 7 | Tumor | Rep. 1 | 756.65 | -0.88 | 101.86 | -0.98 |
|  |  | Rep. 2 | 706.17 | -0.85 | 100.81 | -0.99 |
|  |  | Rep. 3 | 684.28 | -0.86 | 98.54 | -1.00 |
|  | Non-tumor | Rep. 1 | 1105.89 | -0.90 | 153.51 | -1.00 |
|  |  | Rep. 2 | 944.03 | -0.89 | 154.19 | -1.00 |
|  |  | Rep. 3 | 911.52 | -0.85 | 164.49 | -0.99 |
| 8 | Tumor | Rep. 1 | 422.13 | -0.94 | 144.04 | -0.99 |
|  |  | Rep. 2 | 437.24 | -0.95 | 140.33 | -1.00 |
|  |  | Rep. 3 | 424.58 | -0.93 | 148.80 | -0.99 |
|  | Non-tumor | Rep. 1 | 546.63 | -0.94 | 170.60 | -0.99 |
|  |  | Rep. 2 | 533.81 | -0.96 | 173.92 | -0.99 |
|  |  | Rep. 3 | 504.27 | -0.92 | 176.73 | -0.98 |
| 8 | Tumor | Rep. 1 | 532.27 | -0.96 | 190.87 | -1.00 |
|  |  | Rep. 2 | 544.88 | -0.94 | 174.95 | -0.99 |
|  |  | Rep. 3 | 493.22 | -0.98 | 187.95 | -0.99 |
|  | Non-tumor | Rep. 1 | 677.66 | -0.92 | 236.85 | -0.99 |
|  |  | Rep. 2 | 663.84 | -0.94 | 213.23 | -1.00 |
|  |  | Rep. 3 | 705.36 | -0.95 | 212.55 | -0.99 |
