# Supplementary material for: Evaluating T1/T2 Relaxometry with OCRA Tabletop MRI System in Fresh Clinical Samples: Preliminary Insights into ZEB1-Associated Tissue Characteristics
Source: Technol Cancer Res Treat. 2025 Aug 26;24:15330338251366371. doi: 10.1177/15330338251366371 (PMC12381451; doi:10.1177/15330338251366371)
Supplement: sj-docx-3-tct-10.1177_15330338251366371 - Supplemental material for Evaluating T1/T2 Relaxometry with OCRA Tabletop MRI System in Fresh Clinical Samples: Preliminary Insights into ZEB1-Associated Tissue Characteristics [file sj-docx-3-tct-10.1177_15330338251366371.docx]

| Scanning parameter | T1 mapping | | T2 mapping | |
| --- | --- | --- | --- | --- |
|  | Tumor | Non tumor | Tumor | Non tumor |
| LARMOR Frequency [MHz] | 10.380651 | 10.376951 | 10.391051 | 10.384651 |
| TS [ms] | 20 | 20 | 20 | 20 |
| TR [ms] | 6000 | 6000 | 4000 | 4000 |
| TI start [ms] | 10.0 | 10.0 | - | - |
| TI stop [ms] | 2000.0 | 2000.0 | - | - |
| TE start [ms] | - | - | 30 | 30 |
| TE stop [ms] | - | - | 1000.0 | 500.0 |
| TE steps | - | - | 20 | 20 |
