# Supplementary material for: Evaluating T1/T2 Relaxometry with OCRA Tabletop MRI System in Fresh Clinical Samples: Preliminary Insights into ZEB1-Associated Tissue Characteristics
Source: Technol Cancer Res Treat. 2025 Aug 26;24:15330338251366371. doi: 10.1177/15330338251366371 (PMC12381451; doi:10.1177/15330338251366371)
Supplement: sj-docx-4-tct-10.1177_15330338251366371 - Supplemental material for Evaluating T1/T2 Relaxometry with OCRA Tabletop MRI System in Fresh Clinical Samples: Preliminary Insights into ZEB1-Associated Tissue Characteristics [file sj-docx-4-tct-10.1177_15330338251366371.docx]

| Patient Number | Gender | Age | Tumor type |
| --- | --- | --- | --- |
| 1 | Female | 44 | PPPD, Pancreatic carcinoma |
| 2 | Female | 56 | Pancreatic mucinous cystic neoplasia |
| 3 | Male | 55 | Pancreatic NET, Grading G1, pT2, L0, V0, Pn0, R0 |
| 4 | Female | 69 | PDAC, pancreas tail |
| 5 | Male | 64 | CCC |
| 6 | Female | 57 | HCC |
| 7 | Male | 94 | CRC, H1 T1 |
| 8 | Female | 63 | Colorectal adenocarcinoma, Grading G2, pT3, p0N, L0, N0 |
| 9 | Male | 81 | CRC, H1 T1 |
