# Supplementary material for: Evaluating T1/T2 Relaxometry with OCRA Tabletop MRI System in Fresh Clinical Samples: Preliminary Insights into ZEB1-Associated Tissue Characteristics
Source: Technol Cancer Res Treat. 2025 Aug 26;24:15330338251366371. doi: 10.1177/15330338251366371 (PMC12381451; doi:10.1177/15330338251366371)
Supplement: sj-docx-5-tct-10.1177_15330338251366371 - Supplemental material for Evaluating T1/T2 Relaxometry with OCRA Tabletop MRI System in Fresh Clinical Samples: Preliminary Insights into ZEB1-Associated Tissue Characteristics [file sj-docx-5-tct-10.1177_15330338251366371.docx]

**Supplementary Data**

Supplementary Fig. 1: Histological analysis of PDAC, CRC and liver carcinoma samples using H&E staining

Supplementary Table 1: T1 and T2 relaxation times for individual patient samples (n = 9), including both tumor and non-tumor tissues, measured in triplicate. Corresponding R-values from curve fitting are also provided to assess the goodness of fit.

Supplementary Table 2: Acquisition parameters used for T1 and T2 relaxation measurements with the OCRA Tabletop MRI system.

Supplementary Table 3: Demographic and clinicopathological characteristics of the study cohort (n = 9), including patient identification number, gender, age at diagnosis and histopathological classification of tumor type.
